# Regulatory and other rheumatoid factors in rheumatoid arthritis patients with active disease or in remission

**DOI:** 10.1002/jcla.24187

**Published:** 2021-12-24

**Authors:** Liubov Beduleva, Alexandr Sidorov, Kseniya Semenova, Zhanna Khokhlova, Daria Menshikova, Tatyana Khramova, Igor Menshikov

**Affiliations:** ^1^ Laboratory of Molecular and Cell Immunology Department of Immunology and Cell Biology Udmurt State University Izhevsk Russian Federation; ^2^ Laboratory of Biocompatible Materials Udmurt Federal Research Center UB RAS Izhevsk Russian Federation; ^3^ Rezhevskaya Central Regional Hospital Rezh Russian Federation; ^4^ Peoples’ Friendship University of Russia (RUDN University) Moscow Russian Federation

**Keywords:** disease activity, pathological rheumatoid factor, regulatory rheumatoid factor, rheumatoid arthritis

## Abstract

**Background:**

Previously, we identified a regulatory rheumatoid factor (regRF), the production of which provides rats with resistance to collagen‐induced arthritis (CIA). Immunization with conformers of IgG Fc fragments carrying epitopes specific to regRF reduces symptoms of CIA. The aim of this study was to determine whether there is a link between regRF levels and rheumatoid arthritis (RA) activity in humans in order to assess the potential of regRF as a therapeutic biotarget in RA. The variability of rheumatoid factor (RF) specificities present in the blood of RA patients was also studied.

**Methods:**

The regRF were studied in RA patients with active disease and in remission. Variability in the specificities of RF associated with RA was studied by concurrent inhibition of RF latex fixation by variants of modified IgG.

**Results:**

Patients in remission had regRF levels higher than in healthy subjects. The regRF in remission was characterized by tight binding to its antigen, as in healthy subjects. The regRF levels in patients with active RA varied dramatically, and regRF binding to its antigen was weak. The exacerbation of Still's disease coincided with low regRF levels and affinity, while an improvement in patient condition was associated with an increase in regRF levels and affinity. The RF specific to RA, which was detected by the RF latex‐fixation method, was a nonhomogeneous population of antibodies that included RF to lyophilized IgG, to IgG immobilized on polystyrene, and to rabbit IgG.

**Conclusion:**

Stimulating regRF production might enable improved RA therapy.

## Graphical abstract

Patients with active RA were found to have low regRF levels and affinity, while patients in remission had regRF levels higher than in healthy subjects, and the regRF was characterized by tight binding to its antigen. The exacerbation of Still's disease coincides with low regRF levels and affinity, while an improvement in condition is associated with an increase in regRF levels and affinity. The RF specific to RA, which is detected by the RF latex fixation method, is a nonhomogeneous population of antibodies that includes RF to lyophilized IgG, to IgG immobilized on polystyrene, and to rabbit IgG.

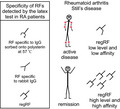

## 1 INTRODUCTION

Despite numerous studies, the only properties definitively established for rheumatoid factor (RF) are that RF is an antibody to the Fc portion of modified IgG, and that elevated RF levels are a diagnostic marker for rheumatoid arthritis (RA). Both the mechanisms by which RF level is elevated in autoimmune and infectious diseases and the role that RF plays in healthy and disease states remain speculative.^1^, ^2^ The accumulated data on RF indicate that the specificity and function of rheumatoid factors are variable. There have been attempts to classify RF into physiological RF, present in the healthy state, and pathological RF,^3^, ^4^ but these have not been brought to fruition. The diversity of methods that researchers have used to detect RF has contributed substantially to the inability to sort out RF variability, as it is not known which rheumatoid factor a particular method detects.

In our own studies of rat resistance and sensitivity to collagen‐induced arthritis, we have found that both arthritis resistance and remission are associated with the production of a rheumatoid factor detected by the agglutination of tanned IgG‐loaded erythrocytes.^5^ We subsequently found a link between disease resistance and RF production in rat models of both experimental autoimmune encephalomyelitis and atherosclerosis induced by immunization with native lipoproteins.^6^ Furthermore, rat arthritis was reduced when arthritic rats were immunized with conformers of IgG Fc fragments bearing epitopes specific to the RF detected by the agglutination of tanned IgG‐loaded erythrocytes.^7^ Therefore, the rheumatoid factor detected by agglutination of tanned IgG‐loaded erythrocytes was named regulatory rheumatoid factor (regRF).^7^ Investigation of the regRF mechanism of action in a rat model of experimental autoimmune encephalomyelitis showed that regRF kills antigen‐activated CD4 T lymphocytes^8^; this may enable regRF to inhibit autoimmune responses.

A comparison of the specificity of regRF with that of the RF associated with human RA indicated that these two RFs are antibodies with different specificities.^9^ Both rheumatoid factors are directed at modified human IgG, but to different epitopes thereof.^9^ Furthermore, the method of agglutination of tanned IgG‐loaded erythrocytes was shown to detect regRF specifically, while the latex test used to detect RF in RA patients detected both regRF and RA‐associated RF, the latter of which is not detected in healthy subjects.^9^

This new information that there are at least two RF populations that differ in their specificity and function, and that both RF populations are present in the blood of RA patients, makes it necessary to take another look at measurements of RF in RA patients. In particular, this raises the question of whether there is a connection between RA activity and the levels of regRF, as well as other rheumatoid factors. Given that immunizing rats with conformers of Fc fragments specific to regRF was effective in suppressing collagen‐induced arthritis, an examination of regRF levels in RA patients with different disease activity levels may reveal whether the lymphocytes that produce regRF can be viewed as a therapeutic biotarget that can be stimulated with conformers of Fc fragments to improve RA therapy.

## 2 MATERIALS AND METHODS

### 2.1 Sera of rheumatoid arthritis patients and donor sera

Sera of rheumatoid arthritis patients were provided by the Rezhevskaya Central Regional Hospital (Rezh, Russia), where patients with rheumatoid arthritis were being followed up or treated. Data were de‐identified. Any experimental investigation with human subjects reported in the manuscript was performed with informed consent and following all the guidelines for experimental investigation with human subjects. Healthy human sera were obtained at the Republic Blood Transfusion Station (Izhevsk, Russia). The sera were used anonymously.

### 2.2 Measurement of RF by the latex‐fixation method

The RF direct latex test (VedaLab, France) was used to detect rheumatoid factor in the sera of arthritis patients.

### 2.3 Measurement of regRF

The regRF titer was determined in an agglutination test using human IgG‐loaded tanned human erythrocytes. For this, group O human erythrocytes (Republic Blood Transfusion Station, Izhevsk, Russia) were fixed with 1% glutaric dialdehyde. The erythrocytes were washed and then incubated for 10 min with tannin solution in PBS at RT. For sensitization, 4 ml of PBS (pH 6.4), 1 ml of a solution of 0.5 mg/ml normal IgG from human serum in 0.9% NaCl, and 70 μl of the pelleted tanned erythrocytes were mixed. Incubation lasted for 20 min at RT; the erythrocytes were washed with 0.9% PBS containing 0.2% BSA. Twofold serial dilutions of serum were prepared and put into wells in aliquots of 50 μl. The same amount of human IgG‐loaded 1.5% erythrocyte suspension was then added. Agglutination results were read after 3 h.

### 2.4 RegRF depletion from rheumatoid arthritis sera

Rheumatoid arthritis sera taken in a volume of 100 μl was mixed with 100 μl of a 7% suspension of tanned IgG‐loaded erythrocytes. The mixture was incubated for 1 h at 37°C on an orbital shaker. The erythrocytes were separated by centrifugation at 260 *g* for 5 min. The resulting supernatant was used. Serum regRF levels before and after depletion were compared by agglutination of tanned IgG‐loaded erythrocytes.

### 2.5 Lyophilized human IgG

Lyophilized human IgG was used to prepare an agglutination test system for detecting regRF and investigating the specificity of patient sera for IgG immobilized on plastic at an elevated temperature. To obtain lyophilized IgG from human plasma, plasma proteins were precipitated with ammonium sulfate (final concentration in solution 210 g/L) and reprecipitated twice with polyethylene glycol 4000 (final concentration in solution 150 g/L). Quarantined plasma was provided by the Republic Blood Transfusion Station (Izhevsk, Russia). Next, size exclusion chromatography was performed using a Sephacryl S 100 26/400 column. An AKTA purifier UPC (GE Healthcare), Spectrophotometer Genesys 10S UV‐Vis (Thermo Fisher Scientific, Inc.), and freeze dryer (FreeZone, Labconco) were provided by the Center for the Collective Use of Scientific Equipment, Udmurt State University, for producing lyophilized human IgG.

### 2.6 Reactivity of RF to human IgG sorbed onto plastic at elevated temperature in RA patients

Lyophilized human IgG produced in our laboratory as indicated above was reconstituted with PBS to its original volume and immobilized on a hydrophobic polystyrene surface of a Corning‐Costar plate at 57°C for 24 h in a quantity of 50 µg/well. Next, the plate wells were washed and blocked with BSA solution. The sera of RA patients (50 µl) was placed into the wells; the sera were used in several dilutions, including the last dilution that induced agglutination of latex particles. The sera were incubated for 1 h at 37°C. The sera that had been incubated with IgG sorbed onto polystyrene were then carefully removed and mixed with 50 μl of a suspension of IgG‐coated latex particles (RF latex test). These same sera incubated at 37°C in plate wells blocked with BSA served as the control. The latex‐fixation results were evaluated after 2 min. A decrease in RF level in the latex test indicated that the RF was reactive toward the immobilized IgG.

### 2.7 Reactivity of RF toward lyophilized human IgG and rabbit IgG in RA patients

To elucidate the specificity of RF to rabbit IgG and human lyophilized IgG, the reaction of concurrent inhibition of latex fixation by rabbit IgG (Equitech‐Bio) and a commercial preparation of human lyophilized IgG (Equitech‐Bio), respectively, was used. The RF titer in rheumatoid arthritis serum was determined by latex fixation (VedaLab). Next, 25 μl of a solution of lyophilized IgG (100, 30 and 10 μg per well) or 25 μl of rabbit IgG (30 μg per well) was added to dilutions of 25 μl of serum, including the titer dilution. As a control, 25 μl of PBS was added in place of the proteins. The solutions were incubated for 1 h at 37°C and then mixed with 50 μl of a suspension of IgG‐coated latex particles (RF latex test). The reaction was read after 2 min.

### 2.8 Statistical analysis of the data

Statistical comparison of data sets was done by Wilcoxon matched pairs test or *t* test or ANOVA using Prism (GraphPad Software).

## 3 RESULTS

### 3.1 RegRF in rheumatoid arthritis patients

A total of 32 patients with an established diagnosis of rheumatoid arthritis were studied (Table 1). Of those, 22 patients had high to moderate rheumatoid arthritis activity. Several of the patients had systemic manifestations—anemia, rheumatoid nodules, Sjögren's syndrome. The duration of disease in the sample of RA patients studied was 2–20 years, with a mean of 8 ± 5.9 years. A total of 35% of patients were receiving combination therapy of a conventional DMARD with a steroids, 31% were being treated with steroids, 12.5% were receiving conventional DMARDs, and 9% were receiving combination therapy of a biologic DMARD with a conventional DMARD and steroids. The remaining 12.5% of patients were not on any treatment at the time of the study and were in remission.

**TABLE 1:** Patients

Patient #/sex	RA duration, years	Therapy	Systemic manifestations	Current state
1/Female	12	Steroids	‐	Active, worsening after COVID−19
2/Female	20	Methotrexate	‐	Active
3/Male	3	Triple therapy	‐	Remission
4/Female	3,5	No, methotrexate was canceled 8 months ago	‐	Remission
5/Female	4	No, methotrexate was canceled 3 months ago	‐	Remission
6/Female	18	Methotrexate	‐	Remission
7/Male	6	Methotrexate, steroids	‐	Active
8/Male	13	Methotrexate	Rheumatoid nodules	Active
9/Male	3,5	Steroids	‐	Active
10/Male	14	Steroids	Anemia	Active
11/Female	2,5	Steroids	‐	Active
12/Male	6	Steroids	‐	Remission
13/Male	5	Methotrexate, steroids	‐	Active
14/Male	14	Methotrexate, steroids	‐	Active
15/Male	2,5	Methotrexate, steroids	‐	Remission
16/Male	11	Methotrexate, steroids	‐	Active
17/Male	7	Methotrexate, steroids	‐	Active
18/Male	19,5	Methotrexate, steroids	‐	Active
19/Female	20	Sulfasalazine, steroids	Sjögren's syndrome	Active
20/Female	1,9	Methotrexate, steroids	‐	Remission
21/Female	10	Methotrexate, steroids etanercept	Diabetes	Remission
22/Male	6	Methotrexate, steroids, sarilumab	‐	Active
23/Male	14	Methotrexate, steroids	‐	Active
24/Male	5,5	Methotrexate, steroids Rituximab	‐	Active
25/Male	3	No, methotrexate was canceled 24 months ago	‐	Remission
26/Male	5	Methotrexate, steroids	‐	Active
27/Female	2	No, sulfasalazine was canceled 6 months ago	‐	Remission
28/Male	11	Steroids	‐	Active
29/Female	14,5	Steroids	‐	Active
30/Male	4	Steroids	‐	Active
31/Female	9	Steroids (first 5 years methotrexate)	‐	Active
32/Female	1,9	Steroids	‐	Active

Of the 32 patients, ten met the ACR/EULAR definition of RA remission on the basis of the number of swollen and painful joints, C‐reactive protein levels, and patient global assessment.^10^ Six of ten were in remission while being treated with conventional DMARD with steroids (three patients), conventional DMARD alone (one patient), steroids alone (one patient), or combination therapy of a biologic DMARD with a conventional DMARD and steroids (one patient). Four of ten patients had gone into remission after treatment (methotrexate or sulfasalazine). But at the time of the study and for 3–24 months preceding it they had been receiving no treatment. These four patients in remission were diagnosed with RA 2–4 years prior. Although these four patients were in remission, they remained RF‐latex‐positive, while none of the 28 healthy subjects studied were positive for RF in the latex test.

**FIGURE 1:**
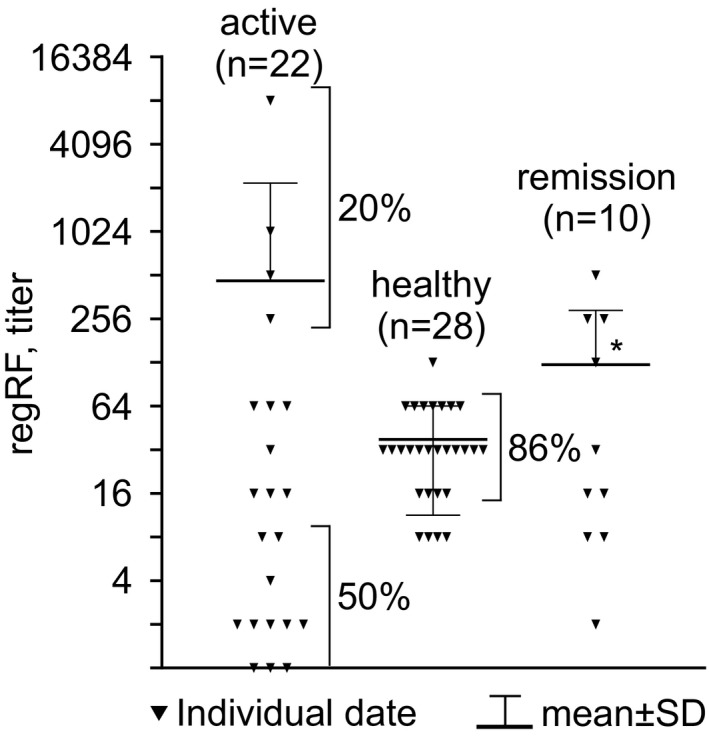
RegRF level detected by agglutination of tanned IgG‐loaded erythrocytes in patients with active RA, patients in remission, and healthy subjects. The results are presented as means ± SD. * ‐ Statistically significant in relation to group “healthy” *p* ≤ 0.05 (*t* test)

A comparative analysis of regRF level in the sera of healthy subjects, RA patients with active disease, and RA patients in remission is shown in Figure 1. RegRF level was higher in patients in remission than in healthy subjects (*t* test) (Figure 1). The mean regRF level in the studied sample of patients with active disease was not significantly different from that of healthy subjects or patients in remission (*t* test) (Figure 1). At the same time, Figure 1 shows that in patients with active disease, the regRF titer varies to an extreme degree, from 0 to 1:8192. Half of the patients with active RA have a relatively low regRF titer, from 0 to 1:8, while 20% of the active patients have an extremely high regRF level, from 1:128 to 1:8192 (Figure 1). For comparison, in 86% of the healthy subjects, the regRF titer is in a narrow range, from 1:16 to 1:64 (Figure 1). Thus, 70% of the RA patients with active disease have regRF levels in their blood that are almost never encountered in healthy subjects.

Next, the tanned IgG‐loaded erythrocyte binding effectiveness of regRF from RA patients and healthy subjects was analyzed. The binding effectiveness of regRF was assessed by the level of regRF that remained in the serum after being incubated with tanned IgG‐loaded erythrocytes that were then precipitated by centrifugation.

**FIGURE 2:**
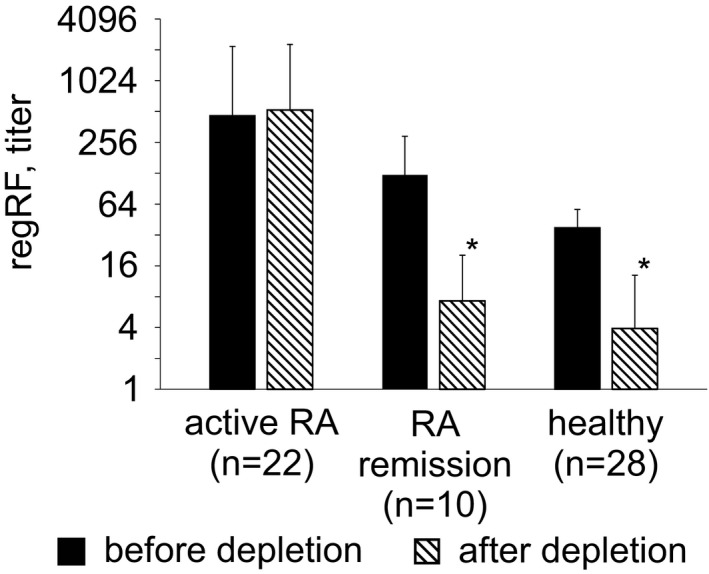
Depletion of regRF from sera of RA patients using tanned IgG‐loaded erythrocytes. The results are presented as means ± SD. * ‐ Statistically significant, *p* ≤ 0.05 (paired *t* test)

As Figure 2 shows, regRF is readily removed from healthy sera. Thus, in 90% of the serum samples, regRF was completely removed from the serum. RegRF from RA patients in remission was completely removed from 80% of the sera (Figure 2). The high levels of regRF in patients in remission also decreased. Furthermore, it was noted that the sera of patients in remission that contained high levels of regRF caused the tanned IgG‐loaded erythrocytes to clump during the incubation, forming visible agglomerates of IgG‐loaded erythrocytes. We suggest that the cause of this phenomenon in patients in remission may be the high affinity of regRF to its epitopes on the IgG. In the patients with active RA, the regRF level remained unchanged after incubation with tanned IgG‐loaded erythrocytes (Figure 2). Considering that regRF in such patients is capable of binding with tanned IgG‐loaded erythrocytes in the plate wells during regRF measurement, but does not bind with the same erythrocytes during the depletion procedure, where the incubation of sera with erythrocytes is performed under constant stirring and requires subsequent centrifugation, we can suggest that in active RA, regRF has low affinity. Therefore, low affinity may be a characteristic of regRF produced in large quantities in patients with active RA that distinguishes it from regRF in patients in remission.

To sum up the resultant data on regRF in RA patients and healthy subjects, we can conclude that in RA patients in remission, regRF is higher than in healthy patients, and the regRF in remission is characterized by tight binding to its antigen, as was found in healthy subjects. In patients with active RA, regRF levels varied widely, from below‐normal to extremely high, and the regRF had low affinity. The low regRF levels in 50% of the patients with active RA and the low regRF affinity, including in 20% of cases where regRF levels are extremely high, suggest that one of the causes of RA and of high RA activity is the low production and/or low affinity of regRF. Since the effect of regRF is based on the killing of activated CD4 lymphocytes,^8^ it is likely that the low regRF production and affinity in RA are not sufficient to keep the expansion of lymphocytes autoreactive to joint antigens in check. These facts point to an important role for regRF in controlling RA and allow us to suggest that stimulating regRF production might enable improved RA therapy.

**FIGURE 3:**
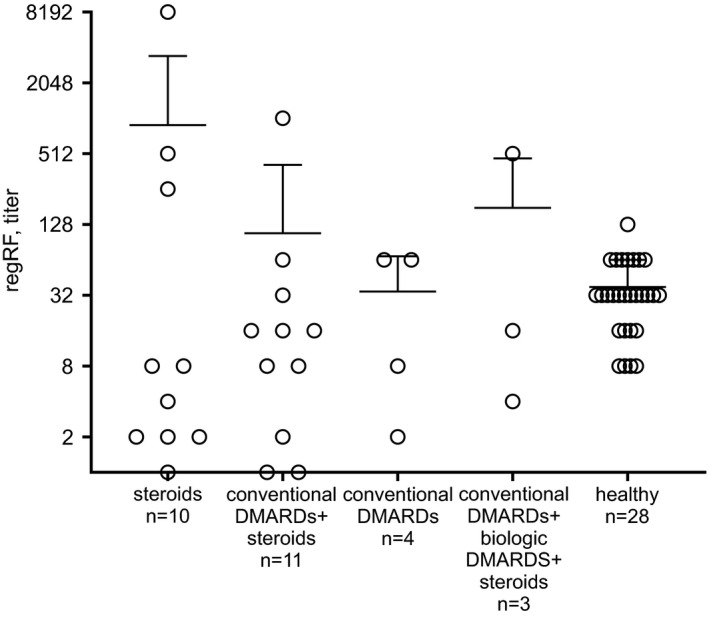
RegRF levels in RA patients receiving various treatments. The results are presented as mean ± SD

A comparison of regRF levels in the blood of RA patients receiving various types of treatment identified no differences (ANOVA) (Figure 3). The “conventional DMARDs” and “biological DMARDs” groups contained few patients, hence the absence of differences can be confirmed only among the “steroids”, “conventional DMARDS + steroids”, and “healthy” groups.

### 3.2 RegRF level during improvement of patient condition in adult‐onset Still's disease

**FIGURE 4:**
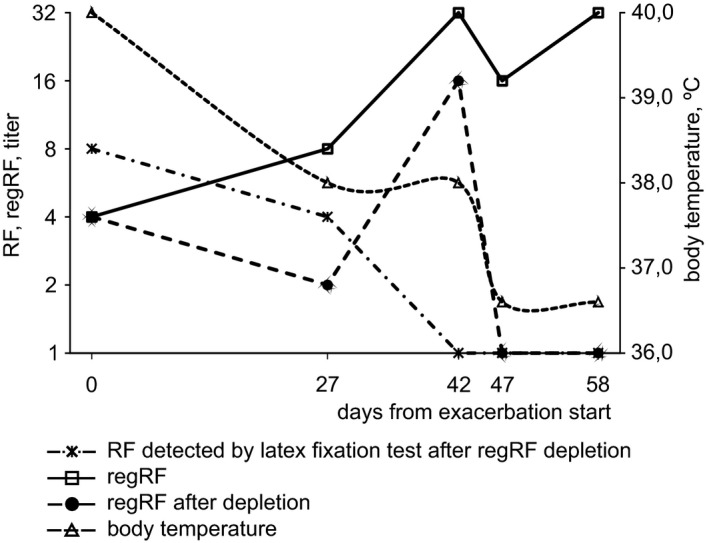
RegRF, RA‐associated rheumatoid factor, and body temperature during improvement of patient condition in adult‐onset Still's disease. RegRF level was detected by agglutination of tanned IgG‐loaded erythrocytes. RA‐associated rheumatoid factor was detected in the RF latex test in the serum depleted of regRF. RegRF was depleted on tanned IgG‐loaded erythrocytes

A male 29 years of age has adult‐onset Still's disease, a rare illness classified as a variant of RA. We were able to follow the regRF level in his blood for 58 days, from the time of a disease exacerbation until his condition improved (Figure 4). The exacerbation involved a fever of 39–40°C and severe pain in the knee joints. The patient was taking methotrexate, sarilumab, alendronic acid, and prednisolone.

The fever and joint pain lasted 42 days from the time the observation commenced (Figure 4). By observation day 47, the temperature had normalized. On day 58, satisfactory condition was noted. Figure 4 shows that at the start of the acute period, RF was detected in the patient's blood by the latex‐fixation method (titer 1:8, which corresponds to 64 IU/µl). Before RF was measured by the latex‐fixation method, the patient serum was depleted of regRF in order to assess the level of RF associated with RA. The blood regRF level at the start of the exacerbation was low (regRF titer 1:4) (Figure 4). Over the subsequent 42 days of observation, the RF level measured in the latex test decreased and ceased to be detected, while the level of regRF increased and reached a plateau. The improvement in the patient's condition that the patient noted starting from observation day 42 of observation, and the disappearance of fever starting from observation day 47, coincided with the regRF reaching a plateau of a 1:32 titer, which is more often detected in healthy subjects (Figure 1).

Figure 4 also shows the results of regRF depletion from the serum of the Still's disease patient. The curve of the serum regRF level and the curve of the regRF level detected in the serum after it was depleted on tanned IgG‐loaded erythrocytes almost completely coincided until observation day 42, which indicates that regRF was not cleared from the serum. However, at the two last observation points, when the patient's condition had substantially improved, regRF had been completely cleared from the serum. Based on these data, we hypothesize that the improved condition of the Still's disease patient was associated not only with an increase in blood levels of regRF, but also with an increase in regRF affinity. Thus, the exacerbation of Still's disease coincides with low regRF levels and likely with low regRF affinity, while the improvement in condition is associated with an increase in regRF levels and affinity. The results from analyzing the clinical case of Still's disease are consistent with the results obtained when patients with active RA were compared with those in remission. When the condition of the Still's disease patient improved, as in RA remission, the property of regRF to tightly bind to tanned IgG‐loaded erythrocytes appears.

The results of the analysis of patients with active RA and RA in remission, as well as the individual clinical case of Still's disease, substantiate the need to intensify regRF production and increase its affinity in RA patients in order to control RA.

### 3.3 Actual level of rheumatoid factor associated with RA in patients in remission

**FIGURE 5:**
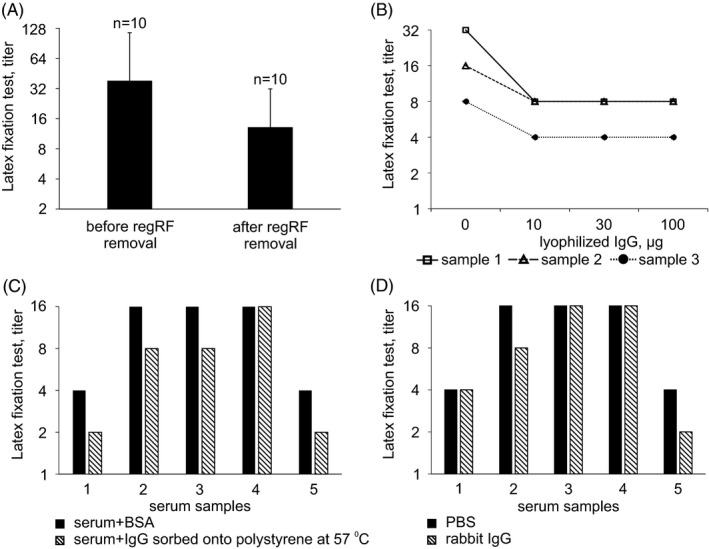
True level and specificity of RA‐associated rheumatoid factor detected in the RF latex test. (A) Rheumatoid factor level (RF latex test) in the serum of RA patients in remission after removal of regRF. (B) Effect of increasing quantities of lyophilized human IgG on latex particle binding of RA patient serum. (C) Effect of RA patient serum depletion by IgG sorbed onto polystyrene on serum latex particle binding. (D) Effect of rabbit IgG on latex particle binding of RA patient serum. B, C, D ‐ all of the sera were first depleted of regRF

Previously we found that in RA patients the RF latex test detects at least two RF populations that differ in their specificity and characteristics.^9^ One of them is regRF, which is an immunoregulatory factor in the healthy state. The other population, which is not detected in healthy subjects, is specific to lyophilized IgG.^9^ Considering that the latex test detects both RF populations, it is not clear how much of the rheumatoid factor associated with rheumatoid arthritis is in the blood of RA patients. To determine this, we removed the regRF from the patient sera and again subjected them to the latex test. Since almost no regRF was removed from the sera of the patients with active RA (Figure 2), the true level of RA‐associated RF could not be determined; therefore, the study of true RA‐associated RF levels was performed in the patients in remission (Figure 5A).

In the group of patients in remission, the RF titer in the latex test decreases by a factor of three after regRF depletion (Figure 5A). Therefore, the true value RA‐associated RF is lower than that detected in whole serum. The antibodies remaining in RA patient serum after removal of regulatory rheumatoid factor can be viewed as associated with RA, since they, in contrast to regRF, are found only in RA patients and not in healthy subjects.

### 3.4 Specificity of RFs associated with RA and detected by the latex‐fixation method

Previously, we showed that antibodies that continue to be detected by the latex‐fixation method in RA patient serum depleted of regRF are specific for lyophilized IgG.^9^ However, we noted that lyophilized IgG does not completely inhibit the RF latex‐fixation reaction induced by RA patient sera. Therefore, as part of our study we tested the effect of increasing quantities of lyophilized IgG on the latex‐fixation reaction of RA patient serum that had been depleted of regRF or did not originally contain regRF (Figure 5B).

We found that adding increasing quantities of lyophilized IgG does not increase inhibition of the latex particle agglutination reaction induced by RA patient serum (Figure 5B). The serum binding curve plateaus when 30 µg of lyophilized IgG is added as a competitor (Figure 5B). It is known that incomplete inhibition of an antibody binding reaction by a competing antigen may be caused by heterogeneity in the subject antibodies.^11^ Since two (samples 1, 3) of the rheumatoid sera studied were first completely depleted of regRF and the third (sample 2) did not contain any regRF from the outset, the observed effect cannot be explained by the presence of regRF that could be detected in the latex test. From this, it follows that in addition to antibodies to epitopes of lyophilized IgG, the serum of RA patients also contains other antibodies to IgG.

The RF reactivity of RA patients to IgG sorbed onto polystyrene at 57°C was studied. For this, the RA patient serum was first depleted of regRF and then incubated in wells of polystyrene plates into which IgG had been sorbed at 57°C. The lyophilized IgG used in these experiments did not inhibit binding of RA patient sera to latex particles. After an hour of incubation with the IgG sorbed onto polystyrene, the patient serum was carefully removed from the wells and again subjected to the latex test. After the sera were incubated with the IgG sorbed onto polystyrene, their titer in the latex test was lower (Figure 5C). Consequently, in the patient serum there are antibodies specific to the IgG that was sorbed onto the plastic at 57°C.

Latex particle binding of some RF‐containing sera was inhibited by rabbit IgG (samples 2 and 5) (Figure 5D).

Therefore, the RF detected in RA patients by the latex‐fixation method is a nonhomogeneous population. We found that the RF from RA patients had affinity to lyophilized IgG, to IgG sorbed onto plastic at 57°C, and to rabbit IgG. All of the diverse rheumatoid factors found can be detected by the RF latex‐fixation method. This may be the reason that the RF latex test has become the most specific and universal test to detect RF associated with rheumatoid arthritis.

## 4 DISCUSSION

The aim of this study was to determine whether there is a link between regRF level and RA activity. Since regRF was revealed in animal studies to be a potential biotarget that could be stimulated to suppress collagen‐induced arthritis, the presence of a connection between regRF and RA remission may serve as justification for stimulating regRF production in RA patients to improve RA therapy.

A study of regRF in patients with active RA and those in remission showed that in the sample of patients with active RA, regRF levels varied dramatically and were either considerably below normal or unusually high, and regRF binding to its antigen was poor, while in the patients in remission, regRF levels were higher than in healthy subjects, and regRF binding to its antigen was as strong as in healthy subjects. An analysis of regRF levels and affinity in a Still's disease patient also revealed that increased regRF level and affinity were associated with the onset of remission. These data support studies on stimulation of regRF production in RA patients.

The latex‐fixation test has become the classical method of RF detection in RA diagnosis. However, the specificity and properties of the antibodies detected by this method were not entirely clear. We found that this method detects several types of RF in the blood of RA patients, and these types differ in their specificity. They include regulatory RF specific to conformers of IgG Fc fragments, RF specific to lyophilized IgG, RF specific to IgG sorbed onto polystyrene, and RF specific to rabbit IgG. We selected the IgG variants for testing based on an analysis of the literature on RF specificity and detection methods in RA patients. The data we obtained on the diverse specificities of RF in RA patients corroborates the results of various researchers. The data we obtained on the specificity of RF to IgG sorbed onto polystyrene corroborates the results of Maibom‐Thomsen et al.^2^ who proposed the method of IgG sorption onto polystyrene at elevated temperature to obtain a modified IgG that has epitopes to RF and can detect RF in RA patients. The data on the specificity of RF to rabbit IgG are consistent with the results of Michaelsen et al.^12^ who showed that RF, reactive exclusively with rabbit but not human IgG‐determinants, can be detected in the serum of some RA patients.

The biological sense behind the appearance of a spectrum of rheumatoid factors in RA remains unclear. We hypothesize that with insufficient production of regRF as a factor inhibiting the expansion of activated, including autoreactive, lymphocytes, the immune system tries to find lymphocyte clones with specificity similar to that of regRF. Therefore, lymphocytes specific to various IgG epitopes are activated. However, this attempt is ineffective, as the increased level of antibodies to various epitopes of modified IgG does not enable suppression of autoimmune responses.

## 5 CONCLUSION

The RA remission is associated with an increase in regRF levels and affinity. Results of an analysis of a clinical case of Still's disease were consistent with the results obtained when patients with active rheumatoid arthritis were compared with those in remission. Therefore, lymphocytes that produce regRF are a potential therapeutic biotarget, as stimulation of them may improve rheumatoid arthritis therapy.

## CONFLICT OF INTEREST

The authors declare that they have no conflict of interest.

## AUTHOR CONTRIBUTIONS

L.B.: Conceptualization, Methodology, Investigation, Writing, Validation, Supervision. I.M.: Conceptualization, Writing—Review & Editing. A.S., K.S, Z.C., D.M., and T.K.: Investigation.

## Data Availability

Data are available on request due to privacy/ethical restrictions.
